# Sensing Dog Brain Reactions to Smell by AI Speckle Pattern Analysis

**DOI:** 10.1002/jbio.202400501

**Published:** 2025-03-11

**Authors:** Ilana Propp, Marianna Beiderman, Sergey Agdarov, Yafim Beiderman, Maxi Blum, Zeev Zalevsky

**Affiliations:** ^1^ Faculty of Engineering and the Nanotechnology Center Bar Ilan University Ramat Gan Israel; ^2^ Department of Electrical and Computer Engineering University of Michigan Ann Arbor Michigan USA; ^3^ Faculty of Engineering, Ruppin Academic Center Kfar Monash Israel; ^4^ System Engineering Analysis Ramat Gan Israel

## Abstract

Investigation of a dog's brain activity related to their outstanding olfactory capabilities has been a topic of interest among researchers. For this specific study, we identified three areas of the brain that have been shown in previous studies to be relevant during the process of smell discrimination in dogs: the olfactory bulb, hippocampus, and amygdala. We set up a detection structure system based on laser and a camera to capture speckle patterns on the three regions in four dog breeds for smell stimuli: garlic, menthol, alcohol, and marijuana. The results were analyzed using an XGBoost model. Our analysis revealed that the amygdala plays a crucial role in scent differentiation. Our work offers insight into leveraging the features that characterize distinct scents in the canine brain, paving the way for developing a compact device that can interpret and translate a dog's olfactory perceptions for human understanding.

## Graphical abstract

The setup of our experiment is shown on Labrador, Johny, a canine participant of our research. The schematic of the setup features: green laser, digital camera, source of smell, and computer. Regions of the brain that were tested are highlighted. Note that the hippocampus and amygdala are on both sides of the dog's brain.

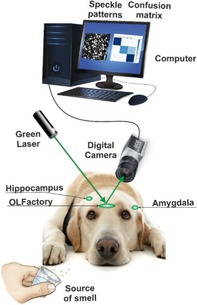

## 1 Introduction

Dogs have an enhanced smelling ability as compared to humans. With a larger olfactory epithelium and nasal cavity, at least three times more olfactory receptor (OR) genes, and unique airflow patterns, dogs can detect a broader range of odorants [1]. Historically, dating back to the 19th century, researchers have utilized various methods to study dog brains, including anatomical dissection, histology, and imaging techniques, leading to significant discoveries. For example, Langley's detailed examination of transverse vertical sections of the dog's brain provided insights into the organization of white and grey matter, the arrangement of the convolutions, and the pathways of olfactory fibers, which are crucial for understanding the advanced olfactory abilities in dogs [2].

### 1.1 Dogs Response to Smell Stimuli in Three Regions of Interest

The olfactory system is a complex and highly specialized sensory network that processes smells from the environment, allowing for odor identification, discrimination, and memory formation. Understanding the trajectory that smells follow through this system provides insight into how organisms perceive and respond to olfactory stimuli. Research has revealed three regions of interest that have a major role in the dog olfactory system—the olfactory bulb, hippocampus, and amygdala.

The olfactory bulb is a crucial relay station in the olfactory system, facilitating the initial processing/filtering and transmission of olfactory information from the nasal cavity to various brain regions. It receives input from olfactory receptor cells (ORCs) projected toward the bulb, which constantly regenerate and possess cilia with surface odor receptors. Each ORC typically contains one type of odor receptor, with the number of receptors per ORC dictating odor intensity [1]. Upon odorant binding, ORs activate G‐protein coupled receptors, initiating a sequence of events that leads to depolarization and the generation of action potentials [1]. The olfactory bulb processes these signals, forming olfactory glomeruli where axons synapse with mitral and tufted cells [3]. Interneurons regulate signal flow, allowing for odor discrimination and detection [4]. In contrast to other species, dogs have significantly larger olfactory bulbs and many more ORCs [1]. In Berns et al.'s study, fMRI analysis revealed the olfactory bulb's significant activation to all scents without differentiating between them [5].

From the olfactory bulb, processed signals are transmitted to primary olfactory cortical regions and further to higher brain areas for odor identification and memory formation. The olfactory bulb communicates with the olfactory cortex in the medial temporal lobes, enabling conscious awareness, odor identification, memory, and localization. In dogs, additional olfactory–cortical tracts, such as the olfactory–occipital tract (OOT) and olfactory–limbic tract (OLT), provide direct links between olfaction and other sensory or cognitive processes. Specifically, the OLT is a large tract looping from the olfactory bulb around the limbic system to the frontal lobe [6].

A part of the limbic system, the hippocampus plays an important role in developing and maintaining short‐term memory. Comprising three highly folded and interconnected main zones, the dentate gyrus, the hippocampus proper, and the subiculum, the hippocampus is located on the medial surface of the temporal lobe. In canines, the hippocampus is 20‐mm‐long and oriented more vertically [7]. Haberly et al., Gottfried et al., and Wilson et al., and Davila et al. found that in dogs that perform odor‐based detection tasks, the hippocampus's role in the olfactory system was related to the development of odor memory within the framework of reward–behavior associations [8, 9, 10, 11]. In addition, Levy et al. observed that dogs with hippocampal lesions suffer from impaired visual and odor recognition memory spans [12]. Furthermore, Dudchenko et al. previously showed that hippocampal lesions have no effect on odor span and odor recognition memory but impair the ability to process and remember a sequence of items such as odors with increasing complexity [13]. Lastly, Jia et al. used noninvasive in vivo fMRI to observe increased activity in brain regions involved in olfactory processing, specifically finding the olfactory bulb and hippocampus particularly active following zinc nanoparticle administration with odorants [14]. They also sought to explore the effect of Weber's Law, which postulates that increasing the amount of an odorant induces a cellular level response. Through this analysis, they found that higher intensity odors triggered increased brain activity in both anesthetized and conscious dogs.

Another member of the limbic system, the amygdala, is associated with emotion processing and memory. Prichard et al. observed through fMRI analysis that the amygdala plays a significant role in processing odors in dogs. They detected neural differentiation between odor stimuli in the amygdala, suggesting that the amygdala is involved in forming associations between specific odors and their outcomes, such as rewards or no rewards. Additionally, the activation patterns in the amygdala suggested that dogs perceive odor mixtures as new odors rather than combinations of their components [15]. The amygdala has also been found to assign emotions to odor profiles, which could contribute to more efficient odor processing [8, 9, 10, 11]. Gläscher et al. found that the left amygdala is involved in more fine‐tuned processing of odor signals from humans to assess threats. This underscores its ability to process scents thoroughly; however, the extent is not fully understood yet [16].

### 1.2 Current Methods to Measure Patterns in Canine Brain's Reaction to Smell

Previous research has explored the primary olfactory structures in dogs but lacks comprehensive knowledge of the connections from the olfactory bulb to the cerebrum. Advanced imaging techniques like fMRI have been used to map the olfactory system in dogs, but these have limitations due to the reliance on highly trained subjects and the activation of top‐down processing regions rather than the olfactory network. However, fMRI has been used to identify several human brain regions involved in odor processing, like the piriform cortex, the amygdala, and the caudate nucleus. Specifically, Berns et al. used fMRI to measure changes in blood flow and oxygenation levels in dog brains to analyze brain activation in response to different olfactory stimuli [4]. In addition, Prichard et al. found fMRI to be useful in tracking indirect indications of neural activation to show how dogs differentiate between mixtures, target odors, and other distracting odors in the amygdala, caudate nucleus, and the olfactory bulb [15]. Another technique that has recently been used is diffusion tensor imaging (DTI), an advanced MRI technique. Andrews et al. used DTI to map the white matter tracts from the olfactory bulb to areas associated with olfactory function. Through this research, they were able to identify five major tracts showing connections to brain regions including the occipital cortex, piriform lobe, limbic system, brainstem, frontal cortex, and entorhinal cortex [6]. Lastly, electroencephalography (EEG) has been used to highlight distinct aspects of cognitive processes in wakefulness, sleep, and comparisons with human EEG studies; however, olfactory processing and cognitive control in dogs are areas underexplored by this technology [17].

Previous studies found olfactory detection thresholds for dogs in the parts‐per‐billion to parts‐per‐trillion (ppt) ranges for various chemical odors. Some studies have reported detection thresholds as low as 1–2 ppt for certain substances like amyl acetate [18, 19]. Studies have used various odor types, including simple chemical compounds, complex odors, and biological scents [18, 19]. However, specific recommended distances for olfactory studies have not been specified, as they likely depend on odor type, concentration, and environmental conditions. Breed type also influences olfactory capabilities; however, there is no conclusive research that specific breeds are more adept than others. Research has identified traits and characteristics that contribute to olfactory success, including the possession of specific OR gene alleles, polymorphism with receptor ligand‐binding capacity, and trainability [2]. Xia et al. performed research to determine how to enhance optical methods to bypass hair and fur‐induced variations. They found that fur has the ability to scatter light; however, it is not capable of blocking the light completely; rather, it diffuses and redistributes parts of the beam. To address this problem, they recommend using lasers with higher wavelengths to penetrate deeper into tissues [20].

### 1.3 Relevance of Speckle Pattern Analysis

Current methods of analyzing dog brain activity, including EEG and fMRI, consider electrical signals caused by synchronized electrical activity of neurons and measure changes in blood flow, respectively. While all mentioned studies rely on complicated ambulatory equipment, a previously unexplored method could offer the opportunity to use remote, non‐invasive technology for analyzing canine olfactory behavior. Laser‐based speckle pattern analysis is one of the modern tools that could be applied for the remote interface with a dog's brain. In the past, this technique has been used for different biomedical applications, such as heart rate detection [21], evaluation of intraocular pressure [22], blood oxygen [23], and neural activity [24]. In considering indications of brain activity, Ozana et al. discovered that speckle pattern analysis effectively measures remote blood pulse pressure through tracking temporal changes in the position and amplitude of secondary speckle patterns reflected in human skin under laser beam illumination [24]. Ozana et al. and Kalyuzhner et al. also found that this photonic‐based method allowed for detecting physiological processes associated with hemodynamic activity by remote monitoring of nanovibrations generated by transient blood flow to the specific regions of the human brain [24, 25]. There is also proof that the laser‐based speckle pattern analysis has been able to penetrate through the skull at specific wavelengths. In the Ozana et al. study, the laser successfully penetrated the skin and skull to a depth sufficient to detect the surface‐level hemodynamic changes in the cerebral cortex [24].

### 1.4 Purpose of This Study

We propose a novel, remote, and affordable technique for dog brain olfactory activity detection, providing a quality answer as to whether it is possible to detect substances by sensing dog brain reactions to smells. We specifically chose the three areas of the brain described above, which can help us further understand the unique olfactory experience that dogs have in comparison to humans. The secondary speckle patterns technique combined with deep neural network data processing methods could aid in using dogs to detect drugs in a variety of situations by recording and analyzing dog brain responses to smells. Since synchronized electrical activity produced by neurons can trigger the release of electrical signals, the process by which a Deep Neural Network algorithm observes nano‐vibrations via speckle pattern analysis is potentially viable if different ORC neurons produce distinct electrical signals after binding to specific smell particles. However, it is important to note that other factors could contribute to differences in speckle patterns, and more research is needed to understand how the recognition of smells might trigger identifiable changes in specific regions of the brain.

## 2 Methods

### 2.1 Speckle Pattern Analyses

When coherent light reflects from a rough surface, the interference of light waves of the same wavelengths and random phase creates speckle patterns containing spots of different intensity. Digital cameras image the plane close to the object; therefore, the amplitude distribution of the speckles equals the Fresnel integral performed over the random phase φ created by the surface roughness. By defocusing the camera, the speckle pattern becomes visible and, despite changing, undergoes horizontal and vertical shifts proportional to the tilting movement of the illuminated surface, while axial and transversal movements do not affect its displacement. The speckle pattern displacement is very sensitive to the surface tilting, allowing the tracking of tiny movements of the illuminated surface [21, 26].

Recording of the speckle pattern video contains numerous frames differing by horizontal and vertical shifts. Cross‐correlation algorithms allow determining the frame shifts and producing a surface displacement graph and vibration frequency distribution after a Fourier transformation. This method, widely applied in numerous biomedical and engineering applications [21, 22, 23, 24, 25, 26, 27], utilizes the requirement for the focal length *F* of the camera, given by [20]:

(1) $$F=\frac{K∆xZ_{3}D}{Z_{2}\lambda }$$

where K is the minimum number of pixels ensuring each speckle is resolved, ∆x is the pixel size, $Z_{2}$ is the distance to the secondary speckle plane from the illuminated surface, $Z_{3}$ is the distance to the camera lens, D is the diameter of the laser spot, and λ is the wavelength of the laser light. To ensure accurate observation, the far‐field approximation requires [21]:

(2) $$Z_{2}>\frac{D^{2}}{4\lambda }$$

This condition ensures that the observed speckle patterns on the camera's detector plane behave predictably as interference patterns in the far‐field region, where the wavefronts are sufficiently planar. The underlying principle of the proposed method lies in using a laser and a defocused fast camera to detect tilting movements, translating them into transversal shifts of the speckle patterns. This approach leverages advancements in machine learning (ML) and deep learning (DL) data processing and classification led to its application to speckle pattern analyses, including the investigation of human brain reaction to stimuli [25]. Our research will be focused on the investigation of the specific dog brain areas under different smell stimuli using the speckle pattern technology combined with DL data processing.

### 2.2 Experimental Setup

**FIGURE 1:**
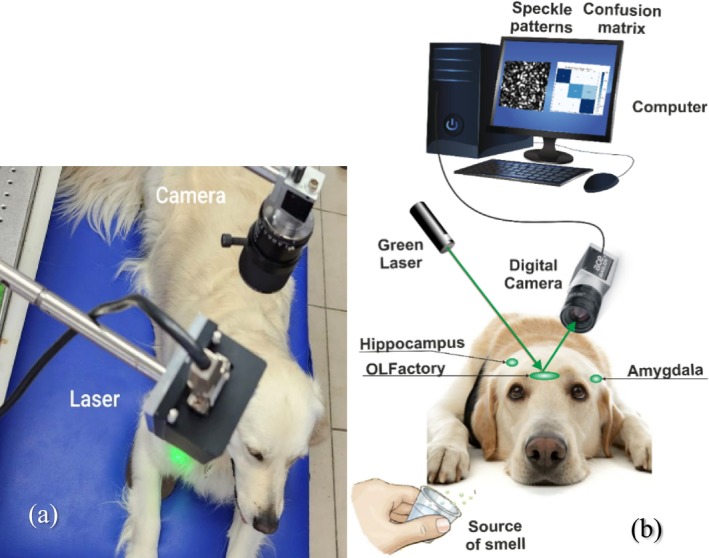
(a) The setup of our experiment is shown on Labrador, Johny (a canine participant of our research). (b) The schematic of the setup features: Green laser, digital camera, source of smell, and computer. Regions of the brain that were tested are highlighted. Note that the hippocampus and amygdala are on both sides of the dog's brain.

The setup for the dog brain investigation contained a 532 nm green laser, Basler digital camera (acA1440‐22um), positioned at 50 cm from the dog's head and computer. The camera was set to capture images at 200 frames per second (FPS) with a resolution of 224 × 224 pixels. The high‐resolution setup allowed for the capture of tiny variations caused by changes in dog's brain activity and blood flow variations related to a smell stimulus, Figure 1. We tested four dogs of different breeds shown in Table 1. Three out of the four dogs are classified as mesocephalic on the CI index, a classification of the ratio of the width of the skull divided by its length. One of the dogs was dolichocephalic. In this experiment, we sought to avoid brachycephalic breeds since they tend to have reduced effectiveness in their nasal capabilities [28]. For the olfactory system stimulation, four smells were chosen based on the similarity of the reactions they elicit but different uses/classifications. Garlic, menthol, alcohol, and cannabis smell sources were placed at 0.1 and 1 m from the dog's nose during speckle pattern recordings under each of the smells applied. The tested dog kept calm, and its eyes were covered with a blindfold. Speckle pattern videos were consequently recorded from the olfactory bulb, hippocampus, and amygdala areas. Each 5‐s recording was repeated four times, resulting in 6000 frames per test for each area.

**TABLE 1:** Tested dog's characteristics.

Dog	Breed	Sex	Age, years	CI classification
Lili	Golden Retriever	Female	3	Mesocephalic
Thomas	Border Collie Great Pyrenees Mix	Male	4	Mesocephalic
Tanin	Papillon	Male	6	Dolichocephalic
Johny	Labrador	Male	6	Mesocephalic

### 2.3 Speckle Pattern Data Processing

The video input data was preprocessed to extract frame sequences of fixed length (224 × 224‐pixel resolution) in chunks of 40 frames with a frame rate of 200 FPS. The frames represented different segments of the videos, capturing the relevant visual and audio features required for classification. From each frame or chunk, various features were extracted using the tsfresh library. These features included positional (pos), differential positional (dpos), spectral centroids, and other relevant time series characteristics. Once the features were extracted, they were loaded into a dataset compatible with the sklearn library along with the corresponding labels.

### 2.4 Model Training

The dataset was split into training, validation, and test sets based on video tags, so that each subset was representative of the overall dataset, helping to maintain the distribution of classes and reducing the risk of overfitting. The training and validation datasets were divided 80%/20% per dog.

An XGBoost (extreme gradient boosting) classifier model was instantiated and configured with initial hyperparameters. The model was trained on the training dataset, using the extracted features and labels. The training process involved iteratively adjusting the model parameters to minimize the classification error on the training data. Optuna, a hyperparameter optimization framework, was used to tune the model's hyperparameters, such as learning rate and tree depth. Optuna uses efficient search algorithms and pruning techniques to explore the hyperparameter space and identify the optimal configuration [29]. This step improved the model's performance by finding the best hyperparameters.

The model's performance was evaluated on the test set, providing an unbiased estimate of the model's generalization ability and helping assess its accuracy and reliability in making predictions on unseen data. Finally, the trained model, along with the feature extraction pipeline, was used to make predictions on new video chunks. This phase involved applying the same preprocessing and feature extraction steps to new data, followed by using the model to predict the corresponding labels.

We focused on secondary, not primary, speckles in this study. Instead of projecting the speckle pattern directly, the pattern was generated due to the roughness of the dog's skin when the laser light was back‐reflected. The size and characteristics of the speckle patterns depends on several experimental parameters, including the size of the illuminating spot and the distance between the laser source, the dog's skin, and the camera capturing the speckles. For this experiment, the illuminating spot size was approximately 2 cm in diameter, and the laser‐to‐skin distance was fixed at 0.5 m.

The experiment was designed to test the model's performance on data from different regions of the brain, each associated with distinct smells. The objective was to determine if the model exhibited higher effectiveness in certain smells and brain regions. We split the smell responses into two categories based on the way we presented the smells to the dogs. Each smell was initially placed at 0.1 m. After taking 3–4 videos, we moved the smell to 1 m, taking three more videos. The inputs for the experiment consisted of videos taken from these regions, categorized into five smell classes: Control (no smell), Alcohol, Marijuana, Menthol, and Garlic. By examining the model's performance across different regions and conditions, we aimed to identify potential patterns in the model's detection capabilities.

### 2.5 XGBoost Model

XGBoost is a powerful machine learning algorithm based on the gradient boosting framework. It is known for its high performance and efficiency in handling datasets of all sizes and complex models [29].

The XGBoost algorithm uses decision tree ensembles, specifically classification and regression trees (CART) where each leaf contains a score, not just a decision algorithm [30]. The data is split from the internal node into leaf nodes that represent a predicted score (outcome). These decision trees are combined into an ensemble, where each tree incrementally corrects the errors of the previous ones, ensuring improved model accuracy.

To understand, XGBoost, it is important to review the basics of supervised learning. In supervised learning, the ML model is defined as a mathematical structure used to predict $y_{i}$ from the input $x_{i}$. The parameters of the model are described as the undetermined parts, θ, that need to be learned from the data. Training supervised learning models involves a process where the best parameters θ are found to fit the training data $x_{i}$ and $y_{i}$. Supervised learning models rely on objective functions to measure how well the model fits the training data, which consists of training loss and a regularization term [30]:

(3) $$\mathrm{obj}\left(\theta \right)=L\left(\theta \right)+\Omega \left(\theta \right)$$

The training loss $L\left(\theta \right)$ measures how well the model's predictions match the actual target values. A common choice of L is the mean squared error for regression tasks of logistic loss for classification tasks. The regularization term $\Omega \left(\theta \right)$ controls the complexity of the model to avoid overfitting. It typically includes terms that penalize large coefficients or complex models [30]:

(4) $$\Omega \left(\theta \right)=\gamma T+\frac{1}{2}\;\lambda \sum_{j=1}^{T}w_{j}^{2}$$

To derive the specific objective function for the XGBoost model, we need to understand the implementation of the algorithm. Since the XGBoost algorithm uses decision tree ensembles, each leaf contains a score based on a decision value. The prediction for a new input is obtained by summing the scores from all trees in the ensemble:

(5) $${\hat{y}}_{i}=\sum_{t=1}^{T}f_{t}\left(x_{i}\right)$$

where $f_{t}$ represents an individual tree in the ensemble. XGBoost approximates the loss function using a second‐order Taylor expansion, which facilitates the optimization of the objective function. The gradient ($g_{i}$) and Hessian ($h_{i}$) terms are derived from the original loss function, which depends on the discrepancy between the predicted values and actual labels [30]:

(6) $$\text{Loss}=\sum_{i=1}^{n}\left[g_{i}f_{t}\left(x_{i}\right)+\frac{1}{2}h_{i}f_{t}^{2}\left(x_{i}\right)\right]$$

where $g_{i}$ is the gradient of the lossfunction and $h_{i}$ is the second‐order (Hessian) derivative of the loss function. These expansions approximate the loss function and are essential for the additive training strategy, where new trees are added sequentially to improve the model.

The optimal weight for a leaf is calculated as [31]

(7) $$w_{j}^{*}=-\frac{G_{j}}{H_{j}+\lambda }$$

where $G_{j}$ is the sum of the gradients for all instances in the leaf, and $H_{j}$ is the sum of the second‐order derivatives (Hessians) for all instances in the leaf. Finally, we can use these two equations to find the full reduction of the objective function after optimization [30]:

(8) $${\mathrm{obj}}^{*}=-\frac{1}{2}\sum_{j=1}^{T}\frac{G_{j}^{2}}{H_{j}+\lambda }+\mathrm{\gamma T}$$

where γ and λ are hyperparameters, T is the number of leaves in tree, and $w_{j}$ are the leaf widgets. The asterisks denote that the values are the optimal values obtained after the optimization process.

The process of building trees additively, where each new tree corrects the errors of the previous ones, ensures that the model incrementally improves and can handle complex patterns in the data. This comprehensive approach ensures that the model not only fits the training data well but also generalizes effectively to new, unseen data.

### 2.6 Hyperparameter Optimization With Optuna

Optuna is a hyperparameter optimization framework that uses a define‐by‐run approach, allowing users to specify complex search spaces that adapt to the structure of the model and the data. Optuna uses an objective function which represents the performance metrics that need to be optimized (e.g., accuracy, *F*_1_ score) [29]. This framework employs various search algorithms, such as Tree‐Structured Parzen Estimator (TPE) and Grid Search, to explore the hyperparameter space. In our code, we use the TPE algorithm to model the distribution of good and bad hyperparameters and use the model to sample new hyperparameters that are more likely to improve the objective function.

### 2.7 Metrics

To evaluate the effectiveness of the experiment, we calculated a confusion matrix. A confusion matrix is a table used to define the performance of a classification algorithm on a set of test data for which the true values are known. We also visualized these results in a normalized confusion matrix, which represents each grouping as having 1.00 samples [32]. It visualizes and summarizes the performance of a classification algorithm by displaying the counts of true positive (TP), true negative (TN), false positive (FP), and false negative (FN) predictions. These four basic characteristics are used to define the measurement metrics of the classifiers [3]:

(9) $$\mathrm{TP}=\text{True positive}=\sum \left(p\left(\mathrm{xi}\right)==1\right)\;\text{and}\;\left(\mathrm{yi}==1\right)$$

(10) $$\mathrm{TN}=\text{True negative}=\sum \left(p\left(\mathrm{xi}\right)==0\right)\;\text{and}\;\left(\mathrm{yi}==0\right)$$

(11) $$\mathrm{FP}=\text{False positive}=\sum \left(p\left(\mathrm{xi}\right)==1\right)\;\text{and}\;\left(\mathrm{yi}==0\right)$$

(12) $$\mathrm{FN}=\text{False negative}=\sum \left(p\left(\mathrm{xi}\right)==0\right)\;\text{and}\;\left(\mathrm{yi}==1\right)$$

The performance metrics of an algorithm were calculated based on the values of TP, TN, FP, and FN. These metrics provide a comprehensive understanding of the classifier's performance [3].

Accuracy is the percentage of accurate predictions, calculated as the ratio of the number of correctly classified instances to the total number of instances. It is defined as

(13) $$\text{Accuracy}=\frac{\left(\mathrm{TP}+\mathrm{TN}\right)}{\left(\mathrm{TP}+\mathrm{FP}+\mathrm{FN}+\mathrm{TN}\right)}$$

Precision is the ratio of positively predicted instances among the retrieved instances, indicating the accuracy of positive predictions. It is defined as

(14) $$\text{Precision}=\frac{\mathrm{TP}}{\left(\mathrm{TP}+\mathrm{FP}\right)}$$

Sensitivity (Recall) is the proportion of actual positives that are correctly identified by the algorithm. It is defined as

(15) $$\text{Sensitivity}=\frac{\mathrm{TP}}{\left(\mathrm{TP}+\mathrm{FN}\right)}$$

Specificity is the proportion of actual negatives that are correctly identified by the algorithm. It is calculated as the number of correct negative predictions divided by the total number of negatives. It is defined as:

(16) $$\text{Specificity}=\frac{\mathrm{TN}}{\left(\mathrm{FP}+\mathrm{TN}\right)}$$

*F*_1_ score provides a balance between precision and sensitivity, offering a single measure of the algorithm's performance. It is defined as [32]

(17) $$\;F_{1}\;\text{score}=\frac{2\times \text{precision}\times \text{recall}}{\text{precision}+\text{recall}}$$

These metrics provide a detailed evaluation of the classification algorithm's performance, highlighting its strengths and areas for improvement. By analyzing these metrics, one can gain insights into the accuracy, reliability, and overall effectiveness of the model.

## 3 Results

The analysis of the model performance across different dogs, smells, and the sensing distances at the three brain regions is presented in this section. By comparing the TP percentages and performance metrics, we sought to identify patterns that could indicate specific regions of the brain where the algorithm was more effective in detecting certain smells. Figure 3 illustrates the TP probabilities per smell tested on each dog, analyzed through the amygdala, hippocampal, and olfactory bulb regions. Figure 4 shows the results we obtained for performance metrics considering the five scores we calculated: accuracy, precision, specificity, f1 score, and sensitivity.

**FIGURE 2:**
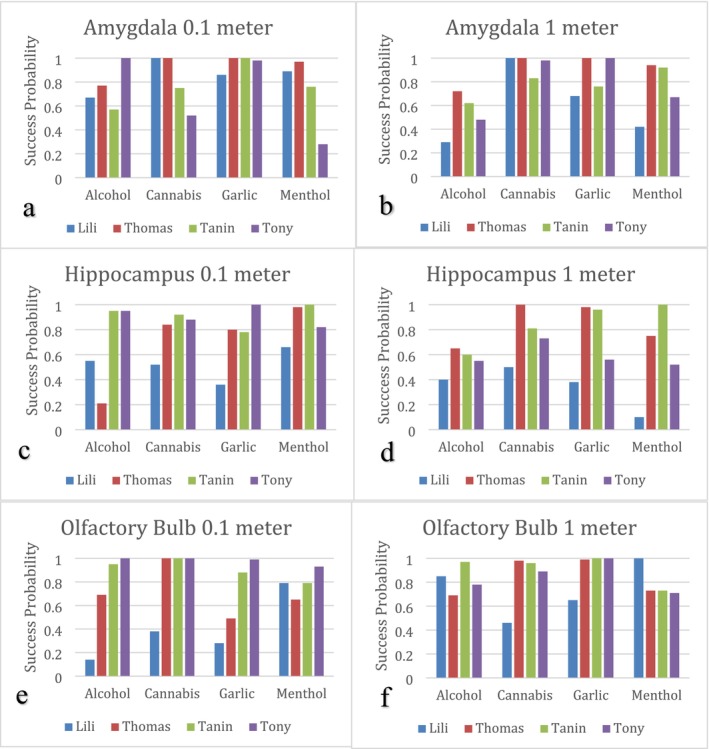
(a, b) Results for the success rate of the normalized confidence matrix for each smell and dog received from the amygdala region of the brain. (c, d) Results for the success rate of the normalized confidence matrix for each smell and dog received from the hippocampus region of the brain. (e, f) Results for the success rate of the normalized confidence matrix for each smell and dog received from the olfactory bulb region of the brain.

The bar charts shown in Figure 2 condense the results taken from the experiment, allowing us to individually analyze the effectiveness of the ML model on each dog, smell, and region of the brain. The success probabilities were derived from the diagonal elements of the confusion matrices produced by our XGBoost algorithm, where each value represents the area where the predicted value matched the actual value for each smell. Starting with the amygdala (Figure 2a), there is clearly a correlation between certain scents, distances, and efficacy. At 0.1 m, the success rate of the algorithm in detecting garlic is markedly higher than that of the other scents, with probability values consistently over 0.80. Another notable trend was observed in the amygdala 1 m distance graph, where the values for cannabis also showed a pattern of success rates above 0.80. The model was most successful at analyzing the speckle patterns for Thomas' amygdala‐region, showing probabilities above 0.95 for every scent except alcohol. Although there is not much conclusive information on which dog breeds excel at smelling, the mix of breeds in his lineage could enhance the number of ORs, facilitating more precise responses transmitted to higher brain regions such as the amygdala. In addition, since it was in the amygdala region, it is possible Thomas has more emotional connections to these specific scents. Moreover, during data collection, Thomas' lighter color made it easier to capture videos, and it is possible his stillness during the specific amygdala data collection part contributed to the quality of the obtained data.

In the hippocampus region (Figure 2b), we detect no clear patterns between dog type, distance, or smell. However, for the olfactory bulb region (Figure 2c), the probability results at 1 m are consistently higher than that at 0.1 m. Specifically, for Lili, Thomas, and Tanin, the overall trajectory of their probability results increased. However, it is hard to find a clear trend for smells and dogs as there is always one exception. For example, in the case of cannabis at 0.1 m, Thomas, Tanin, and Tony all have probability scores of around 1.0; however, Lili's model resulted in a value of 0.38. These discrepancies could be due to systematic errors such as dog movement during testing and accidentally collecting data from a separate region of the brain that was near the olfactory bulb.

**FIGURE 3:**
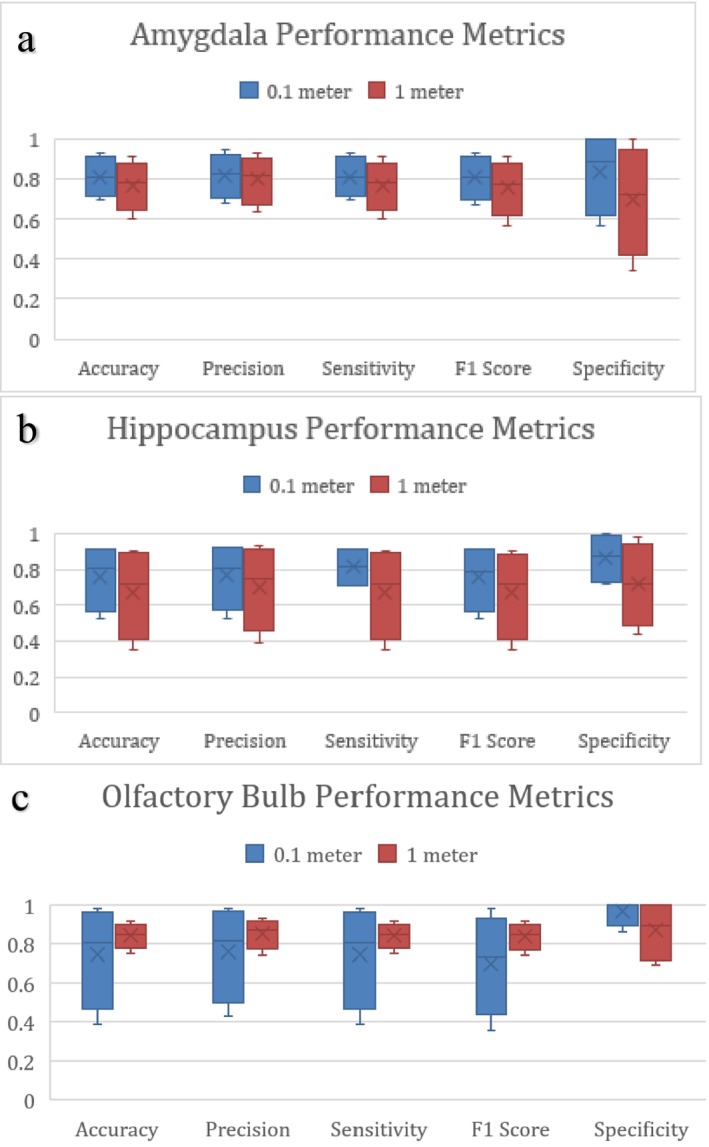
(a) Performance metrics for canine amygdala data at both 0.1‐ and 1‐m distances. (b) Performance metrics for canine hippocampus data at both 0.1‐ and 1‐m distances. (c) Performance metrics for canine olfactory bulb data at both 0.1 m and 1 m distances.

In Figure 3a, the amygdala's first four performance metrics were consistently at or above 0.80, no matter the distance of the smell. The strong correlation between the amygdala and emotional reactions, as suggested by existing research [16], might explain this finding, especially because of high probability values for specific scents like garlic at 0.1 m and cannabis at 1 m, as stated above. We suspect that scents associated with memories and strong emotions could elicit visible reactions, leading to enhanced olfactory differentiation in this region.

We anticipated the hippocampal region to yield the most conclusive results due to its known association with olfactory differentiation, as we observed in our literature review. However, the performance metrics data in Figure 3b revealed that while overall averages for the five metrics were higher, especially for 0.1‐m distances, the overall distribution of the data encompassed a large range. Similarly, the olfactory bulb in Figure 3c values were spread out from probabilities of 0.95–0.40 at 0.1 m. However, at 1 m, the values were more consistent, and the average probabilities were higher than 0.1 m by around 0.05 across the first four metrics. This could be because of the overwhelming nature of the smells we chose, especially when they were placed close to the dog in the area where the receptors would be sending signals to higher regions of the brain, or because of external factors like dog movement and discrepancies in the camera‐laser setup.

**FIGURE 4:**
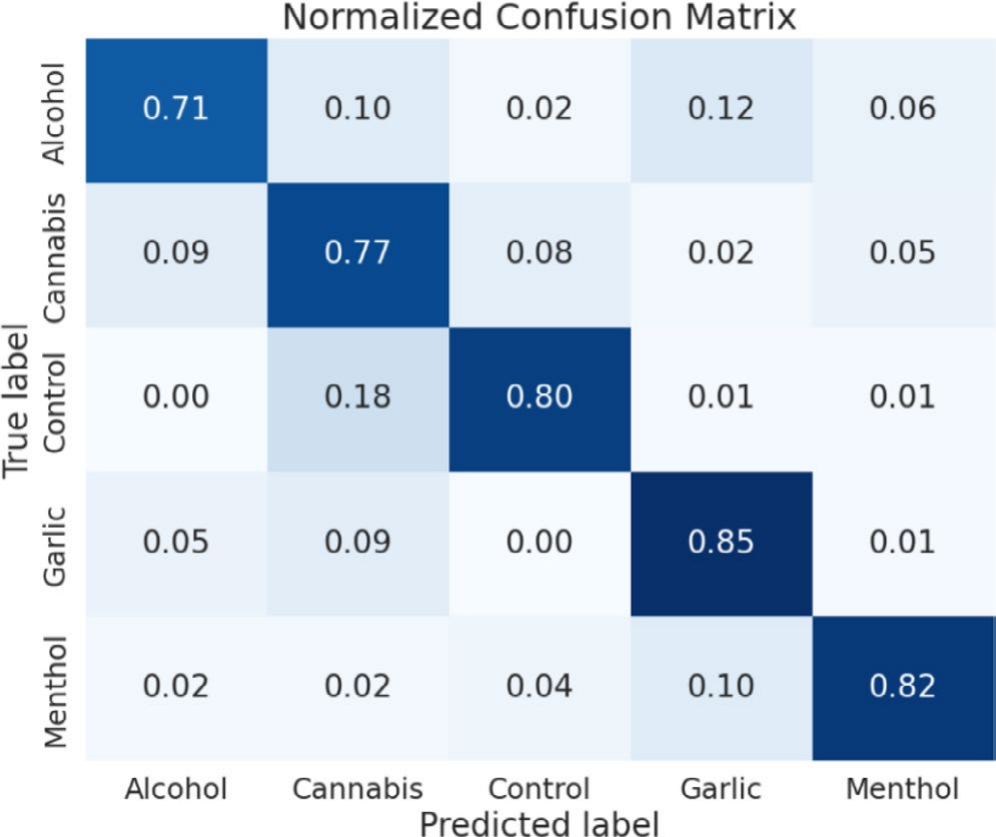
Confusion matrix for canine amygdala data without distinguishing between dog types.

After analyzing the model's performance across all dog types individually, we explored whether aggregating data by brain region rather than dog type would enhance its effectiveness. An important addition to these tests was the inclusion of a control dataset, where no specific smell was presented to the dogs. Previously, we excluded this control from the above data because the results were unreliable and decreased performance metrics, likely influenced by varying human and environmental smells during each testing section. We believed this compromised its role as a reliable control. However, since values for the control in this specific analysis were increased, we decided to include it in Figure 4.

Consistently, the amygdala yielded the most successful results, aligning with our expectations from the analysis above. In Figure 4, the confusion matrix reveals TP scores ranging from 0.71 to 0.85. While our results in Table 2 show performance metrics averaging around 0.80, there is room for improvement in reducing systematic errors. Further experiments aim to address these issues, particularly by scaling up sample size and variety of smells, which we anticipate will boost TP scores and overall performance metrics.

**TABLE 2:** Performance metrics for canine amygdala data without distinguishing between dogs.

Metric	Value
Accuracy	0.79
Precision	0.79
Sensitivity	0.79
Specificity	0.87
*F*_1_ score	0.78

Furthermore, one variable we did not consider was the concentration of smell used. Since we know that dogs have heightened olfactory sensitivity compared to humans, future studies could explore analyzing scent patterns at concentrations below human detection thresholds, potentially enhancing our understanding of scent processing mechanisms. We also did not consider which smells our tested dogs had previously been exposed to and the environments in which these exposures might have occurred to ensure specific smells would not elicit a stronger response.

In summary, these results highlight the potential of specific brain regions, particularly the amygdala, in olfactory detection. Further research could focus on the anatomical and functional characteristics of mixed breeds to better understand their enhanced olfactory capabilities and refine the detection algorithms accordingly. More extensively testing each dog could also facilitate improved classification.

## 4 Discussion and Conclusions

The findings from our experiment lay the groundwork for the development of a novel device aimed at improving our understanding of the olfactory detection capabilities of dogs. We envision creating a compact, Wi‐Fi‐controlled device equipped with a mini camera and laser fixed on the specific dog's scalp area that can efficiently process and analyze olfactory data in real time. This innovative device will feature an embedded LSTM model capable of analyzing video sequences and classifying different smells detected by a dog based on the captured speckle patterns of a dog's brain activity. The device will enhance understanding of a dog's brain reactions to various scents, enabling real‐time detection of drug odors and human diseases through monitoring the canine olfactory system, thereby strengthening the interface between humans and dogs. In our research, a dog's olfactory system is used as a natural smell sensor remotely connected to a computer, allowing our understanding of a particular smell detected.

The obtained results suggest that the Amygdala and the Olfactory Bulb are particularly promising for olfactory detection, given their consistently high scores in accuracy and precision. Further analysis of the amygdala could also provide deeper insights into how olfaction in dogs connects to other brain regions, enhancing our understanding of the neural mechanisms underlying scent detection. More data is needed to make more accurate assumptions on the specific characteristics, study features, and effectiveness of the ML model; however, the study provides a promising direction for the creation of this device.

## Conflicts of Interest

The authors declare no conflicts of interest.

## Data Availability

The data that support the findings of this study are available from the corresponding author upon reasonable request.
